# Role of social support in poststroke depression: A meta-analysis

**DOI:** 10.3389/fpsyt.2022.924277

**Published:** 2022-09-23

**Authors:** Haiyang Bi, Mengjia Wang

**Affiliations:** ^1^Department of Acupuncture, The First Affiliated Hospital of Heilongjiang University of Chinese Medicine, Harbin, China; ^2^Integrated Traditional Chinese and Western Medicine Rehabilitation Medical Center, Heilongjia Provincial Hospital, Harbin, China

**Keywords:** poststroke depression, social support, association, scale, depression

## Abstract

Poststroke depression significantly affects health and quality of life of stroke patients. This study evaluates the role of social support in influencing poststroke depression. The literature search was conducted in electronic databases and study selection was based on precise eligibility criteria. The prevalence rates reported by individual studies were pooled. A meta-analysis of standardized mean differences (SMD) in social support between depressed and non-depressed stroke patients was performed. The odds ratios and correlation coefficients showing the relationship between social support and depression were pooled to achieve overall estimates. Twenty-five studies (9431 patients) were included. The prevalence of depression was 36% [95% confidence interval (CI): 28, 45]. Patients with poststroke depression had significantly lower social support in comparison with patients with no or lower levels of depression [SMD in social support scores −0.338 (95% CI: −0.589, −0.087); *p* = 0.008]. The odds of depression were lower in patients receiving higher levels of social support [OR 0.82 (95% CI: 0.69, 0.95)] but were higher in patients who were receiving weaker social support [OR 5.22 (95% CI: −0.87, 11.31)]. A meta-analysis of correlation coefficients found a significantly inverse correlation between social support and poststroke depression [*r* −0.336 (95% CI: −0.414, −0.254)]. Poststroke depression has a significant independent inverse association with social support.

## Introduction

Stroke, a cerebrovascular accident, occurs when blood flow in the brain is blocked to cause brain damage due to the lack of oxygenation and nutrient supply (1). With over 100 million cases, stroke is the second leading cause of mortality worldwide. Since 1990, the prevalence of stroke has increased by 85% (2). Annually, 15 million individuals suffer from stroke of which ~5 million die, and 5 million become disabled (3). Stroke is associated with the severest and complex disabilities (4). Major risk factors for stroke include hypertension, high body mass index, high fasting blood glucose, environmental particulate pollution, and smoking (2).

Besides causing disabilities of variable degrees, stroke is also associated with comorbidities including cognitive impairment and mood disorders (5). A mental health condition diagnosed within 3 years of stroke is found to be associated with 10% increased risk of mortality after adjustment for other diseases (6). Poststroke depression is associated with higher mortality in the long term (7–9). The prevalence of major depressive disorder and any depressive disorder after stroke are estimated at 17.7 and 33.5%, respectively (10). Depression in stroke patients adversely affects functionality by decreasing motivational thresholds and cognitive abilities. It can affect motor functions, memory, attention, and executive functions (7, 11–13).

Both neurological and psychological components are implicated in the etiology of depression in stroke patients (14). Depression may affect recovery, rehabilitation, the quality of life, and may increase medical care, hospitalization, increased risk of cognitive problems or recurrent stroke, and earlier mortality (15). Several factors are identified to influence poststroke depression. Age and gender are not found to be significantly associated with poststroke depression. Disability, stroke severity, cognitive impairment, apraxia, aphasia, low education level, and social isolation have positive associations with poststroke depression. Diabetes mellitus history may confer an increased risk of poststroke depression. Genetic and epigenetic factors may play a role in poststroke depression (12, 16). Among others, lower education levels, lower income, and sleep disorder are found to be associated with a higher prevalence of poststroke depression (17).

Association of stoke location and depression is not always consistent. Initially, a study observed an association of depression with lesion in the left anterior region (18). Later, a study found that in the first month of stroke dorsally located lesions in the right hemisphere but anteriorly located lesions in the left hemisphere correlated with depression. In this study, there was no correlation between stroke location and depression 1 year after stroke (19). A systematic review published in 2000 found that the risk of poststroke depression was not affected by the stroke location (20). This was also observed in a later review in the overall analysis but in a subgroup of patients with subacute phase (1–6 months poststroke), depression was significantly associated with the right hemisphere (21).

Social support is defined as the exchange of physical or emotional resources between the provider and recipient with an intent to improve the recipient's wellbeing (22). It is the assistance a person receives from his/her social network she/he requires to perform daily life activities, especially in critical situations (23). Sources of social support can be family, relatives, friends, neighbors, and significant others from the community (24). A person's connections within the social network, embeddedness, and affiliation constitute the structural dimension of social support whereas the functional dimension depends on the types of interaction e.g., emotional, instrumental, informational, etc. (25). Besides objective support (actual support), and subjective support (perceived support), the support-seeking behavior of an individual may also affect his/her wellbeing (26).

Social support has been found to be a protective factor against depression in individuals without stroke. Emotional support from parents, spouse, kins, friends, relatives, and other known persons appears to reduce depressive symptoms (27). A positive association is found between social support and the health-related quality of life in stroke patients (28). The high prevalence of depression in stroke patients in the presence of functional disturbances and comorbidities makes it a more serious condition. Several studies have reported the association between social support and poststroke depression. In general, social support is found to have a positive effect on poststroke depression. However, outcomes of various studies are not always consistent which necessitates a systematic review of this area. The objective of the present study was to evaluate the relationship between social support and poststroke depression by conducting a systematic review of the literature and performing meta-analyses of statistical indices to arrive at refined evidence of this association.

## Materials and methods

### Inclusion and exclusion criteria

Studies were included if (a) evaluated the association between poststroke depression and social support; (b) used reliable validated scales to measure depression and social support; and (c) reported statistical indices showing the strength of the relationship between poststroke depression and social support. Exclusion criteria were: studies (a) appraised other related factors such as social activity scales or social health domain of quality of life scales or psychosocial distress measures; (b) did not use validated tool to measure depression; (c) reported descriptive statistics of social support and depression without reporting an association between these; and (d) case reports, theses, and qualitative studies.

### Literature search

The literature search was conducted in electronic databases (Google Scholar, Ovid, PubMed, Science Direct, and Springer). Keywords used for literature search included poststroke depression, social support, emotional support, objective support, subjective support, social network, association, relationship, scale, and inventory. These keywords were used as phrases of logical combinations. After identifying relevant articles, references lists of important articles were also screened for additional studies. The literature search was restricted to original research articles published in the English language before April 2022.

### Data analyses

Demographic, clinical, and pathological data, study design, conduct, and analysis information, depression scales and scores, social support scales and scores, and other related information were extracted from the research articles of the included studies. Quality assessment of the included studies was performed with the Joanna Briggs Institute Critical Appraisal Checklist for Cohort Studies (29). Publication bias assessment was performed with Begg's rank correlation test (30).

Meta-analyses of proportions were performed to estimate the overall percentages of females, married, educational levels, ischemic and hemorrhagic stroke incidences, and the prevalence of poststroke depression. In these meta-analyses, binomial data were used, and the 95% confidence intervals of the estimates were calculated by using score statistics. These meta-analyses incorporated Freeman-Tuckey arcsine transformation for variance stabilization.

Odds ratios depicting the association between social support and depression were pooled under the random-effects model to achieve an overall point estimate by using the DerSimon-Laird method. Meta-analyses of correlation coefficients were performed to achieve an overall correlation between social support and depression in stroke patients. For this purpose, correlation coefficients reported by the individual studies were converted to Fisher's *z*-scores and their respective variance was derived from sample sizes. *Z*-scores were then pooled to achieve overall estimate which was then converted back to the correlation coefficient.

Statistical analyses were performed with Stata software (Stata Corporation, College Station, Texas). All analyses were based on previously published studies, therefore no ethical approval and Informed Consent were required.

## Results

**Figure 1:**
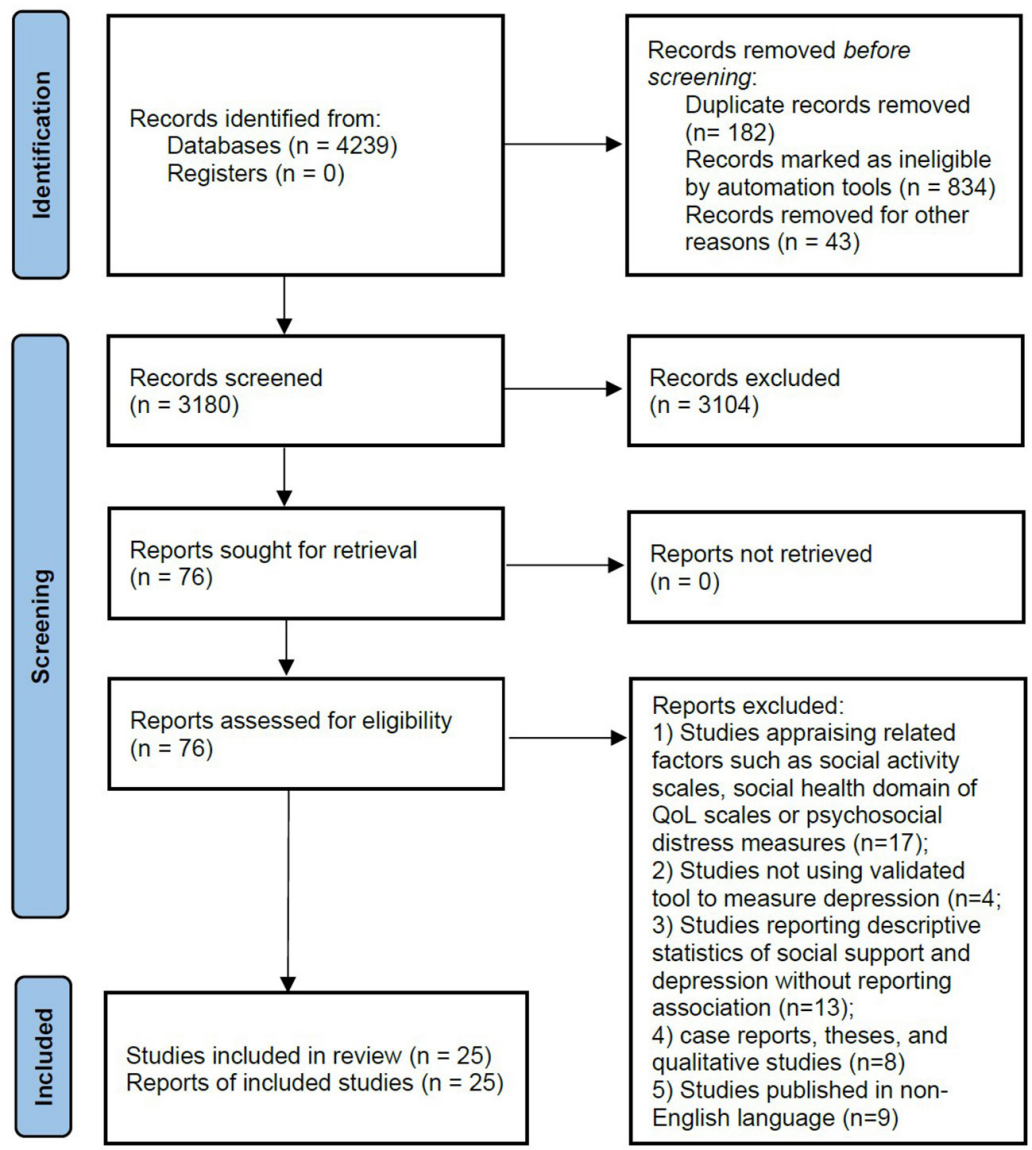
A flowchart of the study screening and selection process.

Twenty-five studies (24, 31–54) were included (Figure 1). In these studies, 9,431 stroke patients were observed. Fifteen of these studies were cross-sectional and nine were longitudinal in design. Proportion of females in this population was 38% [95% confidence interval (CI): 33, 44]. Percentage of married patients was 73% (95% CI: 63, 83). Proportion of patients with primary, secondary, and tertiary education was 38% (95% CI: 27, 49), 36% (95% CI: 24, 50), and 13% (95% CI: 7, 20), respectively. Of this population, 77% (95% CI: 64, 88) had ischemic stroke and 19% (95% CI: 9, 33) had hemorrhagic stroke. Important characteristics of the included studies are given in Supplementary Table S1.

The quality of the included studies was moderate in general (Supplementary Table S2). The major constraint was the lack of information about prestroke depression in patients at the start of the study or at stroke incidence. Among others, whereas nine studies followed patients in a longitudinal design rest of the studies were cross-sectional in design. There was no significant publication bias according to Begg's rank correlation test (adjusted Kendall's score: 1 ± 26.3; *p* = 0.970; Supplementary Figure S1).

**Figure 2:**
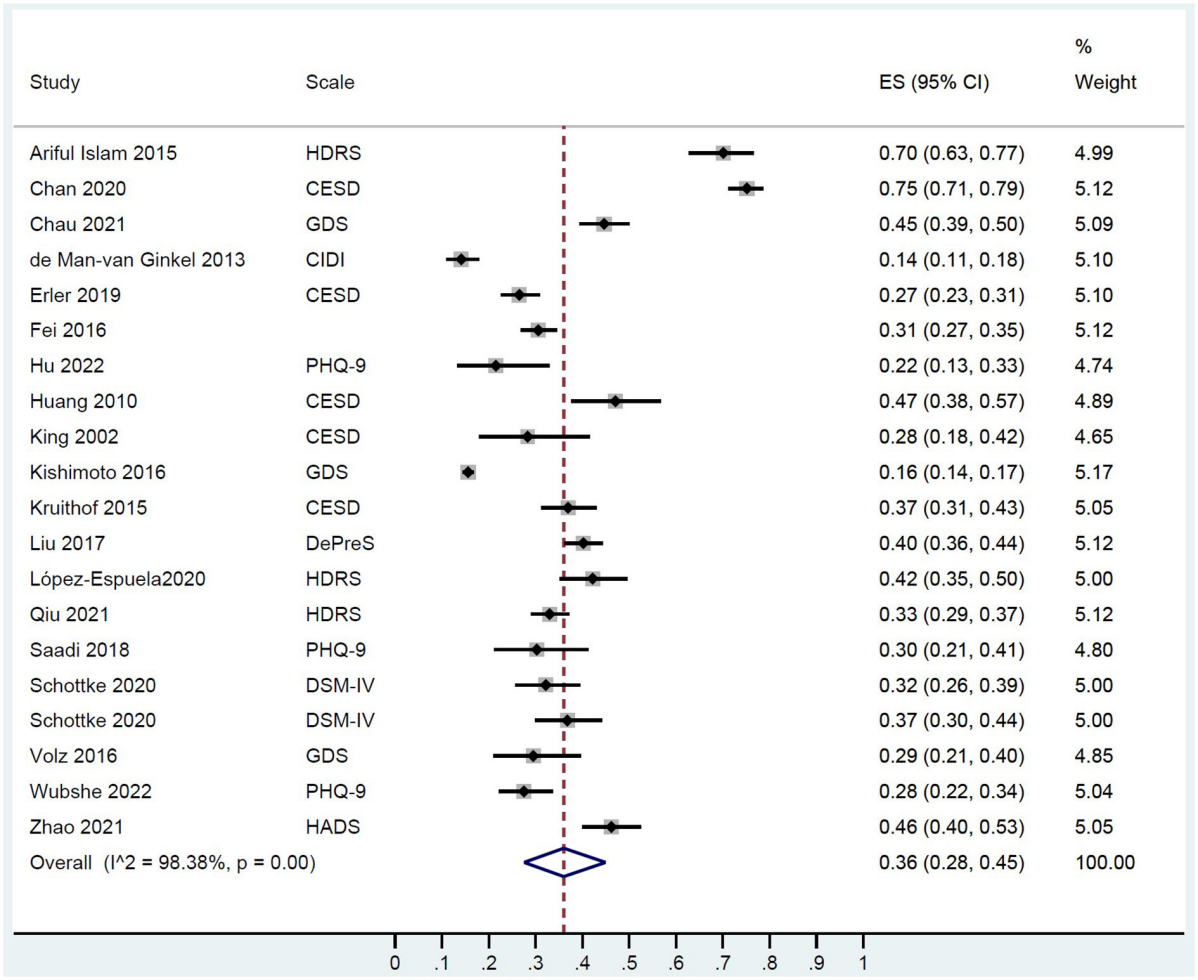
A forest graph showing the prevalence rates of poststroke depression.

The prevalence of depression was 36% (95% CI: 28, 45) in this population (Figure 2). No differences were found between subgroups (< 3 months, 3–12 months, and >1 year after stroke time of depression evaluation) in the prevalence of depression (Supplementary Figure S2).

**Figure 3:**
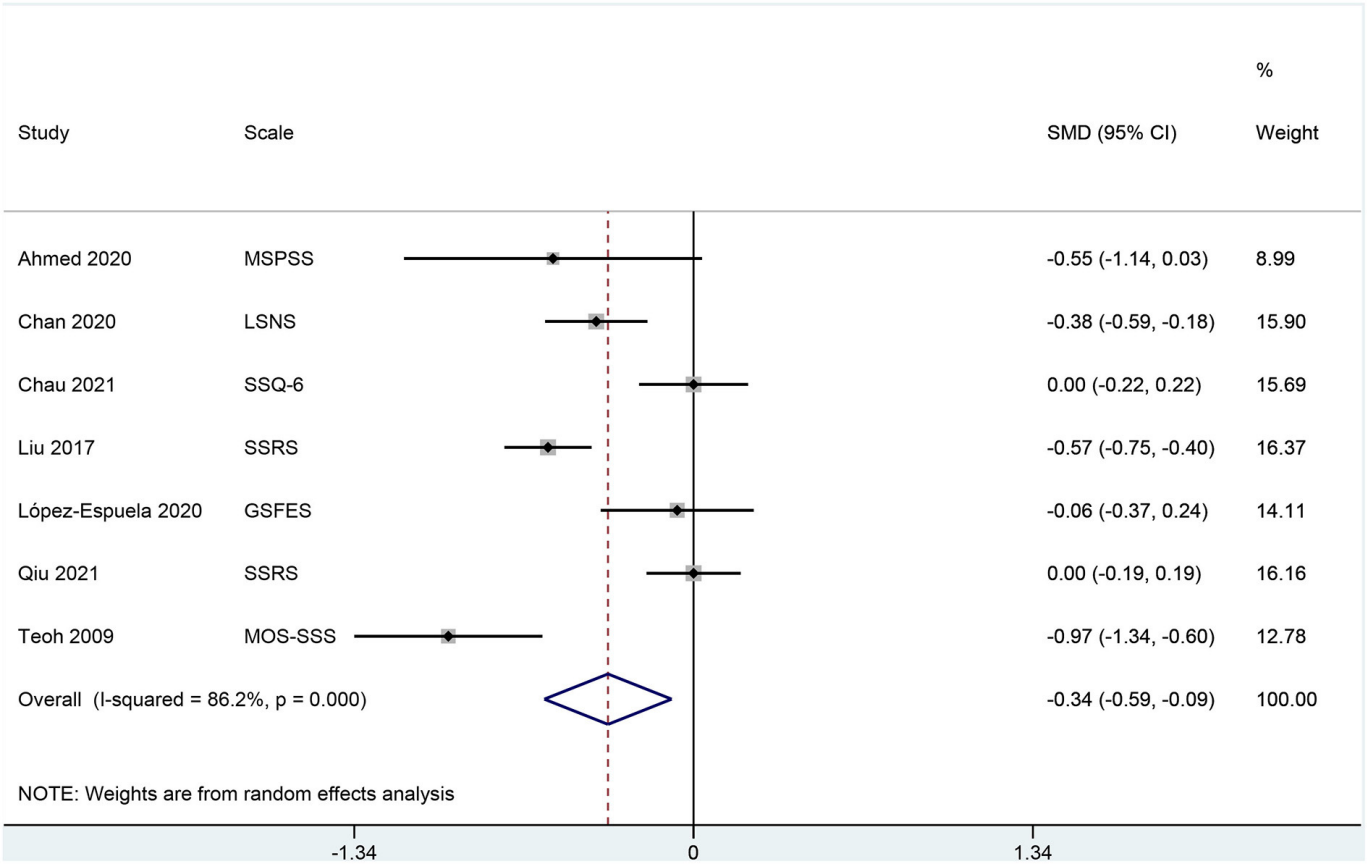
A forest graph showing the outcomes of a meta-analysis of standardized mean differences in social support scores between depressed and non-depressed stroke patients.

**Figure 4:**
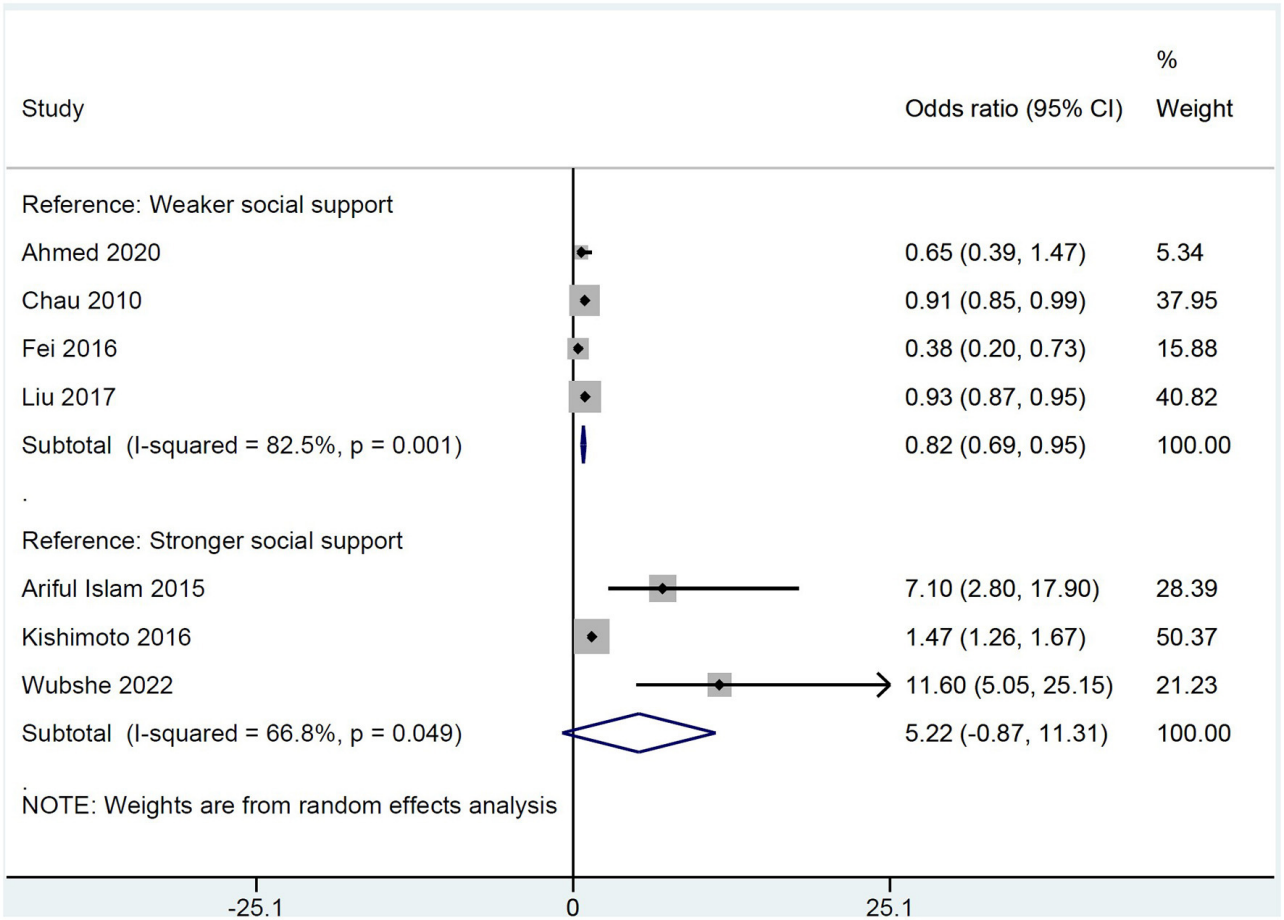
A forest graph showing the outcomes of a pooled analysis of odds ratios depicting the association between poststroke depression and social support.

Social support as measured by any valid scale was significantly lower in patients with poststroke depression in comparison with patients with no poststroke depression [SMD −0.338 (95% CI: −0.589, −0.087); *p* = 0.008; Figure 3]. The odds of poststroke depression were lower in patients receiving higher social support [OR 0.82 (95% CI: 0.69, 0.95)] but were higher in patients who were receiving weaker social support [OR 5.22 (95% CI: −0.87, 11.31); Figure 4].

**Figure 5:**
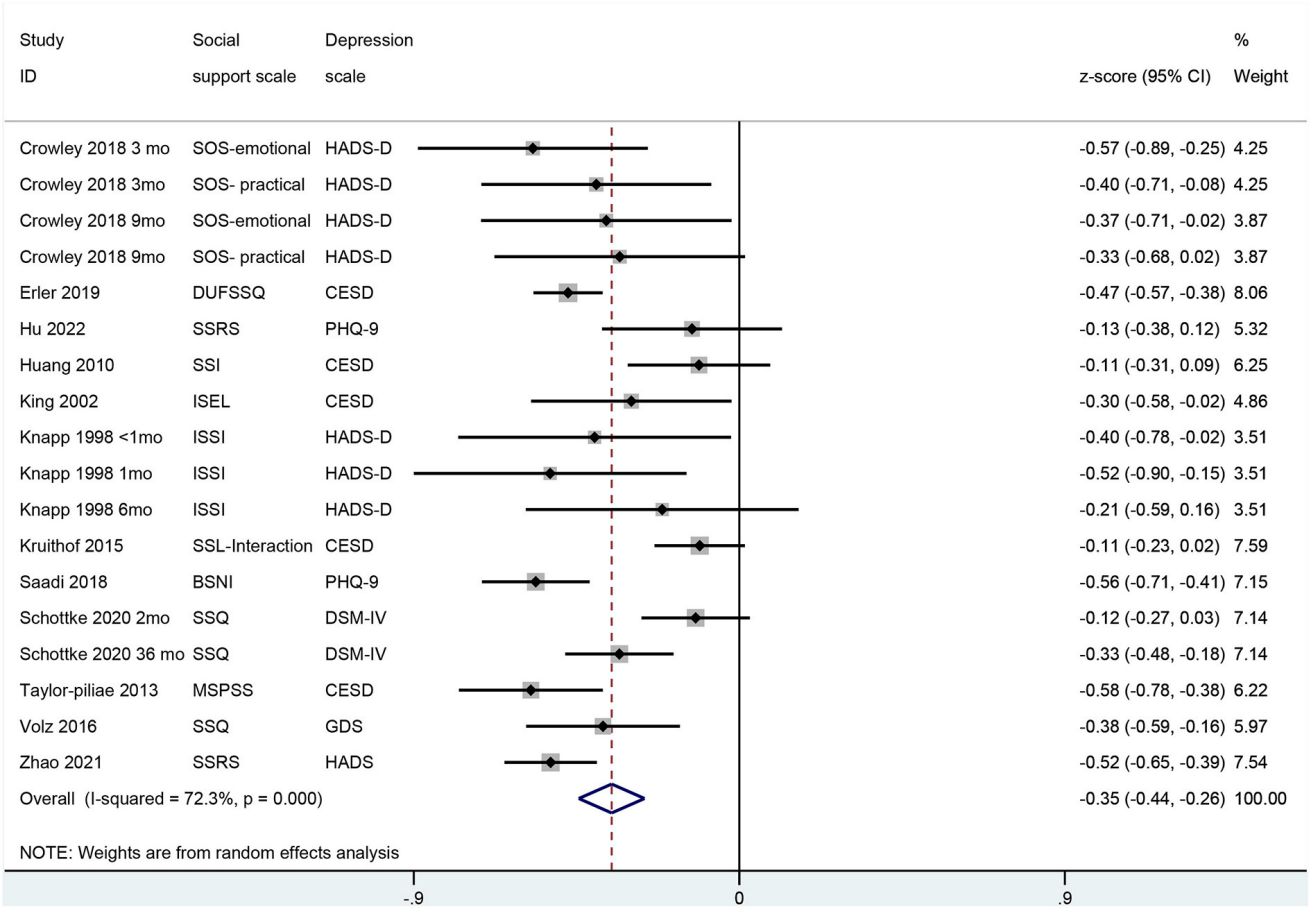
A forest graph showing the outcomes of a meta-analysis of correlation coefficients between poststroke depression and social support.

A meta-analysis of correlation coefficients found significant inverse correlation between social support and depression [*r* −0.336 (95% CI: −0.414, −0.254); Figure 5].

## Discussion

This meta-analysis has found that social support measured by any validated instrument was inversely associated with poststroke depression in (a) a meta-analysis of SMDs in social support between depressed and non-depressed patients, (b) meta-analysis of correlation coefficients between depression and social support, and (c) pooled analysis of odds ratios of social support between depressed and non-depressed patients. The prevalence of poststroke depression was 28–45% in this population.

Many studies that could not be included in the present study also found similar outcomes. Villain et al. (55) found that the extent to which patients were satisfied with the social support they received within 24 h of stroke hospitalization determined depression levels at 3 months poststroke. Social support was inversely associated with poststroke depression in this study. Lewin et al. (56) identified perceived social support as a protective factor against poststroke depression. Lin et al. (57) who provided rehabilitation-related informational support with emotional support including caring, encouragement, and empathy found a negative correlation between social support and poststroke depression. Morris et al. (58) found that if social support was provided only by the spouse was perceived inadequate by the patient and was associated with higher depressive symptoms.

In general, the majority of studies provided evidence suggesting that social support has a protective effect on poststroke depression. However, exceptions do exist. Gottleib et al. (59) found that higher levels of social support were rather associated with increased poststroke depression. However, they also found that lower education level was also positively associated with poststroke depression. Mpembi et al. (60) noted that over 80% of patients with poststroke depression reported unsatisfactory social support. However, after adjustment for age, gender, smoking, alcohol use, and social support, poststroke depression was found to be associated with low-level education status. Kwon et al. (61) who found a significant association between social support and poststroke depression suggested that a lack of social support can be one of the reasons behind poststroke depression, therefore, strategies focusing on improving social support can be fruitful in poststroke depression management.

Prestroke depression may also affect poststroke depression. A meta-analysis of 26 studies found the prevalence of pre-stroke depression to be 11.6% and the presence of prestroke depression increased the odds of poststroke depression (62). Lewin et al. (2013) also identified pre-stroke depression as a strong predictor of poststroke depression. Less studies provided information about pre-stroke depression in the present study. Of the studies included in the present review, Fei et al. (38) noted that among patients with poststroke depression, 38% were using antidepressants before stroke compared to 23% without poststroke depression.

Knapp and Hewison (43) in their stepwise regression found that poststroke depression could be predicted by prestroke and poststroke social support combined. Reid et al. (63) observed that of the patients with prestroke depression, 76% had poststroke depression. On the other hand, of those without prestroke depression, only 26% developed poststroke depression. Prestroke depression was also a predictor of antidepressant medication immediately after stroke (63). A lower degree of recovery is observed for patients with prestroke depression in comparison with those without prestroke depression (64).

Social support components valued by the patients include emotional support (feelings of being loved, and acceptance), tangible support (fostering independence), and the ability to participate (65). The association between social support and poststroke depression is difficult to interpret. Depressed patients may need more than normal familial and societal help and therefore assess the available support as inadequate. Alternatively, depression itself may tend to cause social isolation and altered behavior which can affect rehabilitation and functioning (66). A review focusing on social support and networking in stroke patients found that family support remains consistent for patient although it may involve tensions and disharmony. However, non-familial contacts reduce due to physical disabilities, communication difficulties, fatigue, reduced accessibility, internal barriers, and stigma (65).

Although there exists a relationship between social support and poststroke depression, a review found nine of 10 randomized controlled trials to report the ineffectiveness of social support interventions. However, one RCT that found interventions to be effective differed from others in terms of an earlier start, more intensive scheduling, worker-initiated contact, regular monitoring of depression, and counseling (67). Thus, future studies with better methodologies may clarify the effectiveness of social support programs for alleviating poststroke depression. It can also be valuable to explore in which patients enhanced social support can be more fruitful keeping in view that low education levels, lower socioeconomic status, and prestroke depression pose a higher risk of poststroke depression.

The present study has some limitations. First, the measurements of depression and social support are performed at a considerably wider time ranging from within a month to 39 months poststroke. This can affect the overall outcomes to some extent. The use of various tools for the measurement of depression or social support by the individual studies may also affect the overall outcomes. High statistical heterogeneity observed in the meta-analyses may have roots in the above-given constraints besides others such as patients' characteristics, stroke characteristics, socioeconomic status, and environmental factors. Most of the included study reports lacked data regarding prestroke depression which could provide further important information about the prevalence and management of poststroke depression.

In a population with a prevalence of 36% poststroke depression, this meta-analysis has found that poststroke depression is inversely associated with social support independently. Improving social support for stroke patients can help alleviate depression levels.

## Data availability statement

The original contributions presented in the study are included in the article/Supplementary material, further inquiries can be directed to the corresponding author.

## Author contributions

HB wrote the manuscript. HB and MW collected and analyzed the data. All authors read and approved the final manuscript.

## Conflict of interest

The authors declare that the research was conducted in the absence of any commercial or financial relationships that could be construed as a potential conflict of interest.

## Publisher's note

All claims expressed in this article are solely those of the authors and do not necessarily represent those of their affiliated organizations, or those of the publisher, the editors and the reviewers. Any product that may be evaluated in this article, or claim that may be made by its manufacturer, is not guaranteed or endorsed by the publisher.
