# Supplementary material for: Role of social support in poststroke depression: A meta-analysis
Source: Front Psychiatry. 2022 Sep 23;13:924277. doi: 10.3389/fpsyt.2022.924277 (PMC9539912; doi:10.3389/fpsyt.2022.924277)
Supplement: Supplementary file 1 [file Data_Sheet_1.docx]

| **Table S1: Important characteristics of the included studies** | | | | | | | | | | | | | |
| --- | --- | --- | --- | --- | --- | --- | --- | --- | --- | --- | --- | --- | --- |
| **Study** | **Design** | **n** | **Poststroke months** | **Age (years)** | **Females (%)** | **Married (%)** | **Education status (%)** | | | **IS (%)** | **HS (%)** | **Depression scale** | **Social support scale** |
|  |  |  |  |  |  |  | **Primary** | **Secondary** | **Tertiary** |  |  |  |  |
| Ahmed 2020 [17] | CS | 50 | 3 | 56.7±11.8 | 56 |  |  |  |  |  |  | HADS | MSPSS |
| Chan 2020 [18] | CS | 511 |  | 76.8±6.7 | 48.1 | 67.5 | 35.2 | 51.1 | 13.7 |  |  | CESD | LSNS |
| Chau 2010 [19] | CS | 210 | 6 |  |  |  |  |  |  |  |  | GDS | SSSQ-6 |
| Chau 2021 [20] | CS | 336 |  | 69.9±11.5 | 47 | 78.4 | 28 | 47 | 25 | 80.4 |  | GDS | SSQ-6 |
| Crowley 2018 [21] | L | 41 | 3/9 | 65.7±13.5 | 34 |  |  |  |  | 100 |  | HADS | SOS |
| de Man-van Ginkel 2013 [22] | CS | 382 |  | 67±17 | 31.7 | 63.9 |  |  |  | 87.4 | 12.6 | CIDI | SSL-6 |
| Erler 2019 [23] | L | 422 | 3 | 68.4±13.2 | 48.8 |  |  |  |  |  |  | CESD | DUFSSQ |
| Fei 2016 [24] | CS | 556 |  | 63.5±11.2 | 60 | 33 |  |  |  |  |  |  | None |
| Hu 2022 [25] | L | 65 | 1 |  | 29 | 89 | 23 | 66 | 11 | 80 | 20 | PHQ-9 | SSRS |
| Huang 2010 [26] | CS | 102 | 30 | 64.9±11.8 | 43 | 73.5 | 75 | 22 | 5 |  |  | CESD | SSI |
| Islam 2015 [27] | CS | 164 | 3 |  | 32 | 85 |  | 42.1 | 46.3 | 88 | 17 | HDRS | Social contacts |
| King 2002 [28] | L | 53 | 24 | 60.1±11.8 | 36 | 94.3 |  |  |  |  |  | CESD | ISEL |
| Kishimoto 2016 [29] | CS | 3732 |  | 72±5.4 | 29 |  |  |  |  |  |  | GDS | JMSSSS |
| Knapp 1998 [30] | CS | 30 | <1 | 69±10.5 |  |  |  |  |  |  |  | HADS | ISSI |
| Kruithof 2015 [31] | L | 249 | 12 | 56.7±10.8 | 43 | 72 |  |  |  | 72 |  | CESD | SSL |
| Liu 2017 [32] | CS | 562 | 1 | 64.3±10.4 | 40 |  |  |  |  | 34.7 | 2.3 | DePreS | SSRS |
| López-Espuela2020 [33] | CS | 173 | 6 | 70±12 | 36 | 67 | 70 | 12 | 6 | 89 | 11 | HDRS | GSFES |
| Qiu 2021 [34] | L | 503 | 3 | 58±11 | 23 |  | 27.6 | 56.7 | 15.7 | 90.9 | 9.1 | HDRS | SSRS |
| Saadi 2018 [35] | L | 176 | 3 | 54.4±14.1 | 43 |  | 54 | 11 | 6 | 45.5 | 54.6 | PHQ-9 | BSNI |
| Schottke 2020 [36] | L | 174 | 2/36 |  |  | 65 |  |  |  |  |  | DSM-IV | SSQ |
| Taylor-Piliae 2013 [37] | CS | 100 | 39 | 70±10 | 46 | 59 |  |  | 79 | 68 | 39 | CESD | MSPSS |
| Teoh 2009 [38] | L | 135 | 6 | 67.5±14.3 | 32 | 58.5 |  |  |  | 76.5 | 23.5 | CESD | MOS-SSS |
| Volz 2016 [39] | L | 88 | 6 | 66.4±10.7 | 45 |  |  |  |  |  |  | GDS | SSQ |
| Wubshe 2022 [40] | CS | 229 | 24 |  | 40 |  | 27.5 | 49.3 | 5.7 | 57.2 | 42.8 | PHQ-9 | De Novo |
| Zhao 2021 [41] | CS | 238 |  | 61±9.3 | 32 | 83 |  |  |  | 85.7 | 14.3 | HADS | SSRS |
| **Abbreviations:** CS, cross sectional; HI, hemorrhagic stroke; IS, ischemic stroke; L, longitudinal.  **Abbreviations in Depression scales:** CESD, Center for Epidemiological Studies Depression Scale; CIDI, Composite International Diagnostic Interview; DePreS, Poststroke Depression Prediction Scale; DSM-IV, Diagnostic and Statistical Manual of Mental Disorders-IV; GDS, Geriatric Depression Scale; HADS, Hospital Anxiety and Depression Scale; HDRS, Hamilton Depression Rating Scale; PHQ-9, Patient Health Questionnaire 9.  **Abbreviations in Social Support scales:** BSNI, Berkman-Syme social network index; DUFSS, Duke–University of North Carolina Functional Social Support Questionnaire; GSFEL, Gjon's Social Familial Evaluation Scale; JMSSSS, Jichi Medical School Social Support Scale; ISEL, Interpersonal Support Evaluation List; ISSI, Interview Schedule for Social Interaction; LSNS, Lubben Social Network scale; MOS-SSS, Medical Outcome Study Social Support Survey; MSPSS, Multidimensional Scale of Perceived Social Support; SOS, Significant Other Scale; SSI, Social Support Inventory; SSL, Social Support List 6-Interaction; SSL, Social Support List 6-Perceived lack of support; SSL, Social Support List 6-Perceived excess of support; SSQ, Social support Quaestionnaire; SSRS, Social Support Rating Scale; SSSQ, Social Support Satisfaction Questionnaire 6. | | | | | | | | | | | | | |

| **Table S2: Quality assessment of the included studies** | | | | | | | | | | | | | | | | | | | | | | | | | |
| --- | --- | --- | --- | --- | --- | --- | --- | --- | --- | --- | --- | --- | --- | --- | --- | --- | --- | --- | --- | --- | --- | --- | --- | --- | --- |
| Criteria | Study reference number | | | | | | | | | | | | | | | | | | | | | | | | |
|  | 17 | 18 | 19 | 20 | 21 | 22 | 23 | 24 | 25 | 26 | 27 | 28 | 29 | 30 | 31 | 32 | 33 | 34 | 35 | 36 | 37 | 38 | 39 | 40 | 41 |
| 1. Were the two groups similar and recruited from the same population? | Y | Y | Y | Y | Y | Y | Y | Y | Y | Y | Y | Y | Y | Y | Y | Y | Y | Y | Y | Y | Y | Y | Y | Y | Y |
| 1. Were the exposures measured similarly to assign people to both exposed and unexposed groups? | Y | Y | Y | Y | Y | Y | Y | Y | Y | Y | Y | Y | Y | Y | Y | Y | Y | Y | Y | Y | Y | Y | Y | Y | Y |
| 1. Was the exposure measured in a valid and reliable way? | Y | Y | Y | Y | Y | Y | Y | Y | Y | Y | Y | Y | Y | Y | Y | Y | Y | Y | Y | Y | Y | Y | Y | Y | Y |
| 1. Were confounding factors identified? | Y | Y | Y | Y | Y | N | Y | Y | Y | Y | Y | Y | Y | N | Y | Y | Y | Y | N | Y | Y | N | Y | Y | N |
| 1. Were strategies to deal with confounding factors stated? | Y | N | N | Y | N | Y | Y | Y | N | Y | Y | N | Y | N | N | Y | Y | Y | N | N | Y | N | Y | Y | N |
| 1. Were the groups/participants free of the outcome at the start of the study (or at the moment of exposure)? | U | U | U | U | U | U | U | U | U | U | U | U | U | U | U | U | U | U | U | U | U | U | U | U | U |
| 1. Were the outcomes measured in a valid and reliable way? | Y | Y | Y | Y | Y | Y | Y | Y | Y | Y | U | Y | Y | Y | Y | Y | Y | Y | Y | Y | Y | Y | Y | U | Y |
| 1. Was the follow up time reported and sufficient to be long enough for outcomes to occur? | N | N | N | N | Y | N | Y | N | Y | N | N | Y | N | N | Y | N | N | Y | Y | Y | N | Y | Y | N | N |
| 1. Was follow up complete, and if not, were the reasons to loss to follow up described and explored? | NA | NA | NA | NA | Y | NA | U | NA | U | NA | NA | N | NA | NA | Y | NA | NA | U | U | Y | NA | NR | U | NA | NA |
| 1. Were strategies to address incomplete follow up utilized? | NA | NA | NA | NA | NA | NA | U | NA | U | NA | NA | U | NA | NA | NA | NA | NA | U | U | NA | NA | NA | U | NA | NA |
| 1. Was appropriate statistical analysis used? | Y | N | N | Y | N | Y | Y | Y | N | Y | Y | N | Y | N | N | Y | Y | Y | N | N | Y | Y | Y | Y | N |
| Legends: Y, Yes, N, No; NA, not applicable; U, Unclear | | | | | | | | | | | | | | | | | | | | | | | | | |

Figure S1: A funnel graph showing the outcomes of publication bias test (Begg’s test) using the correlation coefficient between poststroke depression and social support meta-analysis data.

Figure S2: A forest graph showing the depression rates in stroke patients with subgroups of >1 year and <1 year time of depression evaluation.
